# Supplementary material for: Content-rich biological network constructed by mining PubMed abstracts
Source: BMC Bioinformatics. 2004 Oct 8;5:147. doi: 10.1186/1471-2105-5-147 (PMC528731; doi:10.1186/1471-2105-5-147)
Supplement: Additional File 2 — The original results of the above study (non-essential files are deleted to keep the file size under the limit set by BMC bioinformatics). [file 1471-2105-5-147-S2.bz2 › chilibotAdditionalFile2/dip50/585ID9311977E10953/html/RNH_ANG.html]

 


 **RNH** and **ANG** 
  
Found 69 abstracts in PubMed, retrieved 50.  
 

 What does Google say? 
 PDF only 
| .edu only 

---

**Interactive relationship** (e.g. stimulation, inhibition, etc)

**Inhibitory relationship**- Thiol disulfide exchange of ribonuclease inhibitor  [ **RNH** ]  bound to ribonuclease A  [ **ANG** ] .  Ref: 7499372 J Biol Chem, 1995
- Potential modulators of angiogenesis such as protamine, heparin and the placental ribonuclease inhibitor  [ **RNH** ]  competed for angiogenin  [ **ANG** ]  binding to the cells.  Ref: 10103013 Eur J Biochem, 1999
- Basic homopolyamino acids, histones and protamines are potent antagonists of angiogenin  [ **ANG** ]  binding to ribonuclease inhibitor  [ **RNH** ] .  Ref: 10025952 FEBS Lett, 1999
- Anti angiogenin  [ **ANG** ]  antibodies and the angiogenesis inhibitors platelet factor 4 and placental ribonuclease inhibitor  [ **RNH** ]  prevent adhesion of HT 29 cells to angiogenin  [ **ANG** ] .  Ref: 7510698 J Biol Chem, 1994
- A radio ribonuclease inhibitor  [ **RNH** ]  assay based on the interaction of 125I angiogenin  [ **ANG** ]  with ribonuclease inhibitor  [ **RNH** ]  RI was used to detect pancreatic type ribonucleases and potential modulators of their action.  Ref: 10025952 FEBS Lett, 1999
- Complex between bovine ribonuclease A  [ **ANG** ]  and porcine ribonuclease inhibitor  [ **RNH** ]  crystallizes in a similar unit cell as free ribonuclease inhibitor  [ **RNH** ] .  Ref: 8057371 J Mol Biol, 1994
- Residues 287 301 of human placental ribonuclease inhibitor  [ **RNH** ]  have been reported to inhibit pancreatic ribonuclease A  [ **ANG** ]  in a similar way as the entire inhibitor Crevel Thieffry, I.  Ref: 8218387 Biochim Biophys Acta, 1993
- Mechanism of ribonuclease inhibition by ribonuclease inhibitor  [ **RNH** ]  protein based on the crystal structure of its complex with ribonuclease A  [ **ANG** ] .  Ref: 9000628 J Mol Biol, 1996
- RNasin, the placental ribonuclease inhibitor  [ **RNH** ] , is known to have antiangiogenic activity through the inhibition of angiogenin  [ **ANG** ]  and basic fibroblast growth factor.  Ref: 11393280 Cancer Gene Ther, 2001
- The cytosolic ribonucleolytic activity of ribonuclease A  [ **ANG** ]  RNase A and other pancreatic type ribonucleases is limited by the presence of excess ribonuclease inhibitor  [ **RNH** ]  RI.  Ref: 11437592 Protein Expr Purif, 2001
- Human placental ribonuclease inhibitor  [ **RNH** ]  PRI is a potent protein inhibitor of pancreatic ribonucleases and the homologous blood vessel inducing protein angiogenin  [ **ANG** ] .  Ref: 2408043 Proc Natl Acad Sci U S A, 1990
- The ribonuclease inhibitor  [ **RNH** ]  from human placenta is a tight binding inhibitor of alkaline and neutral ribonucleases, including the blood vessel inducing protein, angiogenin  [ **ANG** ] .  Ref: 2276743 Genomics, 1990
- We describe the mechanism of ribonuclease inhibition by ribonuclease inhibitor  [ **RNH** ] , a protein built of leucine rich repeats, based on the crystal structure of the complex between the inhibitor and ribonuclease A  [ **ANG** ] .  Ref: 9000628 J Mol Biol, 1996
- Ribonuclease inhibitor  [ **RNH** ]  RI was purified about 1300 fold from human cerebrum including a small portion of midbrain by a combination of ammonium sulfate precipitation, ribonuclease A  [ **ANG** ]  Sepharose chromatography, and high performance anion exchange chromatography.  Ref: 8037455 Arch Biochem Biophys, 1994
**Neutral relationship**- Genetic screen to dissect protein protein interactions ribonuclease inhibitor  [ **RNH** ]  ribonuclease A  [ **ANG** ]  as a model system.  Ref: 12431438 Methods, 2002
- Similar leucine rich repeats present in a series of other proteins, e.g., the mammalian ribonuclease angiogenin  [ **ANG** ]  inhibitor  [ **RNH** ] , adenylyl cyclase from S. cerevisiae, the toll protein from Drosophila melanogaster, and the sds22 protein phosphatase regulatory subunit from S. pombe, are thought to be involved in protein protein interactions.  Ref: 8374168 Mol Biol Cell, 1993
- The interactions of human placental ribonuclease inhibitor  [ **RNH** ]  PRI with bovine pancreatic ribonuclease RNase A and human angiogenin  [ **ANG** ] , a plasma protein that induces blood vessel formation, have been characterized in detail in earlier studies.  Ref: 1998683 Biochemistry, 1991
- These results represent the first clues concerning specific domains in these proteins and assign them to the LRM superfamily, which includes such members as yeast adenylate cyclase, cell surface protein receptors and ribonuclease angiogenin  [ **ANG** ]  inhibitor  [ **RNH** ] , all exerting their function by specific protein protein interactions involving LRM domains 1988 EMBO J.  Ref: 2044757 FEBS Lett, 1991
- Analysis of the interactions of human ribonuclease inhibitor  [ **RNH** ]  with angiogenin  [ **ANG** ]  and ribonuclease A  [ **ANG** ]  by mutagenesis importance of inhibitor residues inside versus outside the C terminal hot spot.  Ref: 10970748 J Mol Biol, 2000
- Modulation of that activity might involve interaction of angiogenin  [ **ANG** ]  with cell surface receptors and extracellular matrix of endothelial cells, tight binding inhibition of both its ribonucleolytic activity and cell binding property by ribonuclease inhibitor  [ **RNH** ] , as well as the overall influence of divalent copper, a modulator of angiogenesis.  Ref: 1723310 Blood Coagul Fibrinolysis, 1990
- In addition, binding of ARH III to the placental ribonuclease inhibitor  [ **RNH** ]  is increased by at least 1 order of magnitude Ki less than or equal to 7 x 10 17 M compared to angiogenin  [ **ANG** ] .  Ref: 1692238 Biochemistry, 1990

**Non-interactive relationship** (e.g. studied together, co-existance, homology, etc.)

- The yeast RNA1 protein, necessary for RNA processing, is homologous to the human ribonuclease angiogenin  [ **ANG** ]  inhibitor  [ **RNH** ]  RAI  [ **RNH** ] .  Ref: 1603072 Mol Gen Genet, 1992
- Ribonuclease A  [ **ANG** ]  RNase A and the ribonuclease inhibitor  [ **RNH** ]  protein RI form one of the tightest known protein protein complexes.  Ref: 12069420 Anal Biochem, 2002
- Ribonuclease angiogenin  [ **ANG** ]  inhibitor  [ **RNH** ]  RAI  [ **RNH** ]  is a tight binding inhibitor of ribonucleolytic and angiogenic activities involved in tumor progression.  Ref: 1544320 Cytogenet Cell Genet, 1992
- Within this superfamily RNA1 is most closely related to the ribonuclease angiogenin  [ **ANG** ]  inhibitor  [ **RNH** ]  RAI  [ **RNH** ] , which is a tightly binding inhibitor of ribonucleolytic activities in mammals.  Ref: 1603072 Mol Gen Genet, 1992
- Previous single site mutagenesis studies on the complexes of ribonuclease inhibitor  [ **RNH** ]  RI with angiogenin  [ **ANG** ]   **Ang**  and RNase A suggested that in both cases a substantial fraction of the binding energy is concentrated within one small part of the crystallographically observed interface, involving RI residues 434 438.  Ref: 10413501 Biochemistry, 1999
- Three genes were placed on the contig in the following order telomere ribonuclease angiogenin  [ **ANG** ]  inhibitor  [ **RNH** ]   **RNH**  Harvey ras 1 HRAS1 HRAS1 related complex HRC.  Ref: 8661149 Genomics, 1996
- The interaction of rabbit, pig and bovine angiogenins with placental ribonuclease inhibitor  [ **RNH** ] , a potent inhibitor of angiogenin  [ **ANG** ] , was examined by fluorescence spectroscopy.  Ref: 8448182 Biochim Biophys Acta, 1993
- The efficacy of this approach is demonstrated with the ribonuclease inhibitor  [ **RNH** ]  protein and ribonuclease A  [ **ANG** ] , which form a complex with an equilibrium dissociation constant in the femtomolar range.  Ref: 12431438 Methods, 2002
- Polyacrylamide gel electrophoresis and the crystal density indicate that both ribonuclease inhibitor  [ **RNH** ]  and ribonuclease A  [ **ANG** ]  are present in the crystals.  Ref: 8057371 J Mol Biol, 1994
- Molecular recognition of human angiogenin  [ **ANG** ]  by placental ribonuclease inhibitor  [ **RNH** ]  an X ray crystallographic study at 2.0 A resolution.  Ref: 9311977 EMBO J, 1997
- We present here the crystal structure at 2.5 A resolution of the complex between ribonuclease A  [ **ANG** ]  and ribonuclease inhibitor  [ **RNH** ] , a protein built entirely of leucine rich repeats.  Ref: 7877692 Nature, 1995
- Binding of placental ribonuclease inhibitor  [ **RNH** ]  PRI to  **Ang**  also was affected by the nature of the linker and by the presence or absence of a spacer.  Ref: 8555226 Biochemistry, 1996
- We obtained three different morphologies of co crystals of bovine ribonuclease A  [ **ANG** ]  and porcine ribonuclease inhibitor  [ **RNH** ] .  Ref: 8057371 J Mol Biol, 1994
- A recent report placed the gene for placental ribonuclease inhibitor  [ **RNH** ]   **RNH** , ribonuclease angiogenin  [ **ANG** ]  inhibitor  [ **RNH** ]  within 90 kb of HRAS1 by pulsed field gel electrophoresis PFGE mapping.  Ref: 7710782 Genet Anal Tech Appl, 1994
- Superadditive and subadditive effects of hot spot mutations within the interfaces of placental ribonuclease inhibitor  [ **RNH** ]  with angiogenin  [ **ANG** ]  and ribonuclease A  [ **ANG** ] .  Ref: 10413501 Biochemistry, 1999
- Human placental ribonuclease inhibitor  [ **RNH** ]  PRI, a 50 kDa tight binding inhibitor of angiogenin  [ **ANG** ]  and pancreatic ribonuclease, consists predominantly of 7 internal repeats, each 57 residues long.  Ref: 2397204 Biochemistry, 1990
- Fluorescence assay for the binding of ribonuclease A  [ **ANG** ]  to the ribonuclease inhibitor  [ **RNH** ]  protein.  Ref: 12069420 Anal Biochem, 2002
- The recently determined crystal structure of the ribonuclease A  [ **ANG** ]  ribonuclease inhibitor  [ **RNH** ]  complex suggests the basis for the protein binding function of leucine rich repeats.  Ref: 7583641 Curr Opin Struct Biol, 1995
- The human ribonuclease angiogenin  [ **ANG** ]  inhibitor  [ **RNH** ]  is encoded by a gene mapped to chromosome 11p15.5, within 90 kb of the HRAS protooncogene.  Ref: 1544320 Cytogenet Cell Genet, 1992
- Twenty four hour ambulatory blood pressure measurements, plasma renin activity PRA,  **Ang**  II and concentrations of  **RNH**  6270, the pharmacologically active metabolite of CS 866, were monitored up to 24 h after medication.  Ref: 9488244 J Hypertens, 1997
- The nucleotide sequence of the open reading frame shared significant homology with that of the human ribonuclease angiogenin  [ **ANG** ]  inhibitor  [ **RNH** ]  RI.  Ref: 11167794 Br J Haematol, 2001
